# Acute Jaundice in Sheep

**Published:** 1882-01

**Authors:** 


Acute Jaundice in Sheep.—Researches made at the Veterinary School in Berlin have shown that acute jaundice may be produced in sheep by feeding them upon fodder containing lupine (pulse). This jaundice is due to an acute yellow atrophy of the liver, resembling very closely the same disease in man, as it occurs idiopathically or after poisoning with phosphorus.

When the sheep are fed for a long time on specimens in which the poisonous properties are less active, an interstital hepatitis develops.

The urine always contains biliary pigments, generally albumen and often hyaline and granular casts.

It is a remarkable and interesting fact that, in spite of the very great atrophy of the liver, the urine always contains urea or hippuric acid, while leucin and tyrosin are never found. One of the strongest arguments for supposing that the liver breaks up leucin and tyrosin (themselves the products of the decomposition of nitrogenous bodies) into urea and its allies, lay in the fact that, in acute yellow atrophy, urea and uric acid disappear from the urine, while leucin and tyrosin are substituted. The inference has been that the functional activity of the liver being destroyed by disease, that organ could not manufacture urea out of the leucin and tyrosin supplied to it. It is evident that, if the facts above given are correct, some other explanation of the origin of urea, as regards the liver, must be given.

The lupine in question poisons, in a similar manner, the horse, goat and dog.

The toxic principle is insoluble in ether, alcohol and glycerine, is slightly soluble in pure water, and very soluble in alkaline solutions. It is thought to be either an organic acid or a glucoside.

